# Supplementary material for: MetaRibo-Seq measures translation in microbiomes
Source: Nat Commun. 2020 Jun 29;11:3268. doi: 10.1038/s41467-020-17081-z (PMC7324362; doi:10.1038/s41467-020-17081-z)
Supplement: Supplementary file 10 — Supplementary Data 7 [file 41467_2020_17081_MOESM10_ESM.zip › File2/Confidence_High_Taxonomy/36460_out.krona.html]

Javascript must be enabled to view this page.

members
magnitude
magnitudeUnassigned
count
unassigned
taxon
rank

36460\_out

10

10
2
superkingdom

10
1224
phylum

10
class
1236

order
91347
10

10
543
family

10
561
genus

562
species

SRS017191\_contig\_number\_contig-100\_616.116516SRS019068\_contig\_number\_47573SRS019601\_contig\_number\_12901SRS049402\_contig\_number\_12531SRS077646\_contig\_number\_contig-100\_1007.1008SRS140492\_contig\_number\_8067SRS143423\_contig\_number\_contig-100\_7347.101186SRS144362\_contig\_number\_23171SRS146888\_contig\_number\_10921SRS147022\_contig\_number\_12979
10
